# Effects of Renal Denervation on Renal Artery Function in Humans: Preliminary Study

**DOI:** 10.1371/journal.pone.0150662

**Published:** 2016-03-22

**Authors:** Adelina Doltra, Arthur Hartmann, Philipp Stawowy, Leonid Goubergrits, Titus Kuehne, Ernst Wellnhofer, Rolf Gebker, Christopher Schneeweis, Bernhard Schnackenburg, Murray Esler, Eckart Fleck, Sebastian Kelle

**Affiliations:** 1 Deutsches Herzzentrum Berlin, Department of Internal Medicine/Cardiology, Berlin, Germany; 2 Biofluid Mechanics Laboratory, Charité - Universitätsmedizin Berlin, Berlin, Germany; 3 Deutsches Herzzentrum Berlin, Department of Congenital Heart Disease and Paediatric Cardiology, Berlin, Germany; 4 DZHK (German Center for Cardiovascular Research), partner site Berlin, Berlin, Germany; 5 Philips Healthcare, Clinical Science, Hamburg, Germany; 6 Baker IDI Heart & Diabetes Institute, Melbourne, Australia; University Medical Center Utrecht, NETHERLANDS

## Abstract

**Aim:**

To study the effects of RD on renal artery wall function non-invasively using magnetic resonance.

**Methods and Results:**

32 patients undergoing RD were included. A 3.0 Tesla magnetic resonance of the renal arteries was performed before RD and after 6-month. We quantified the vessel sharpness of both renal arteries using a quantitative analysis tool (Soap-Bubble®). In 17 patients we assessed the maximal and minimal cross-sectional area of both arteries, peak velocity, mean flow, and renal artery distensibility. In a subset of patients wall shear stress was assessed with computational flow dynamics. Neither renal artery sharpness nor renal artery distensibility differed significantly. A significant increase in minimal and maximal areas (by 25.3%, p = 0.008, and 24.6%, p = 0.007, respectively), peak velocity (by 16.9%, p = 0.021), and mean flow (by 22.4%, p = 0.007) was observed after RD. Wall shear stress significantly decreased (by 25%, p = 0.029). These effects were observed in blood pressure responders and non-responders.

**Conclusions:**

RD is not associated with adverse effects at renal artery level, and leads to an increase in cross-sectional areas, velocity and flow and a decrease in wall shear stress.

## Introduction

It is estimated that 1 out of 50 patients with newly diagnosed hypertension (HT) will develop resistant HT, which carries an increased risk for cardiovascular and renal complications [1]. In the past years, several trials have demonstrated the usefulness of renal denervation (RD) as a non-pharmacological treatment for resistant hypertension [2–4]. In addition to its blood pressure lowering effect, data from the main RD trials have demonstrated a good safety profile, without significant renovascular complications or renal function impairment at follow-up [2–5]. Despite this safety evidence, however, acute optical coherence tomography data has shown the presence of significant local injury that may be not apparent in angiography [6,7]. The clinical impact of those findings, though, is unknown.

On the other hand, invasive data arising from animal studies suggest that RD could lead to an increase in peak velocity and renal artery flow [8], but to date no human studies have investigated this topic. Magnetic resonance (MR) imaging permits the non-invasive anatomic study of renal arteries and the assessment of hemodynamic parameters related to vessel function [9]. The aim of our study was to assess the effect of RD on renal arteries non-invasively, using state-of-the-art cardiovascular MR techniques.

## Methods

Thirty-two patients with resistant hypertension undergoing RD between April 2012 and November 2013 were prospectively enrolled. Resistant hypertension was defined as an office systolic blood pressure (SBP) above the target (≥140 mm Hg) or mean ambulatory 24-h SBP >135 mm Hg despite the use of ≥3 antihypertensive agents of different classes, including a diuretic at maximum or highest tolerated doses [1]. Blood pressure measurement methods are described in detail elsewhere [10]. A stable antihypertensive medication regime (> 3 month treatment with stable dosage) was necessary before inclusion. One patient with multiple allergies to antihypertensive preparations was also included. Exclusion criteria were contraindications to RD (significant renal artery stenosis, renal arteries with a diameter < 4 mm or a length < 20 mm or presence of multiple renal arteries [11], pseudo-resistant hypertension (mean ambulatory 24-h SBP <130 mm Hg), secondary hypertension, and GFR < 45 ml/min/1.73 m^2^. Patients with general contraindications for the performance of cardiovascular MR were also excluded.

All patients included underwent a MR study at baseline (≤1 week before RD) that was repeated at 6 month follow-up. Blood pressure was determined during both MR exams in order to quantify renal artery distensibility. Clinical assessment, including serum creatinine analysis, review of medication compliance and blood pressure determination according to the Standard Joint National Committee VII Guidelines [12]was also performed at both time points. A Symplicity Flex system catheter (Medtronic, Minneapolis, MN, USA) was used in the RD procedure as previously reported [13], with a mean number of ablation points of 5.7 ± 1.2 (right renal artery) and 5.9 ± 1.0 (left renal artery). A positive response to RD was defined as a reduction of ≥10 mmHg in systolic blood pressure at 6-month follow-up [3]. The study was approved by the local institutional review board (Charité - Universitätsmedizin Berlin) and written informed consent was obtained from all patients before inclusion.

### MR protocol

**Fig 1:**
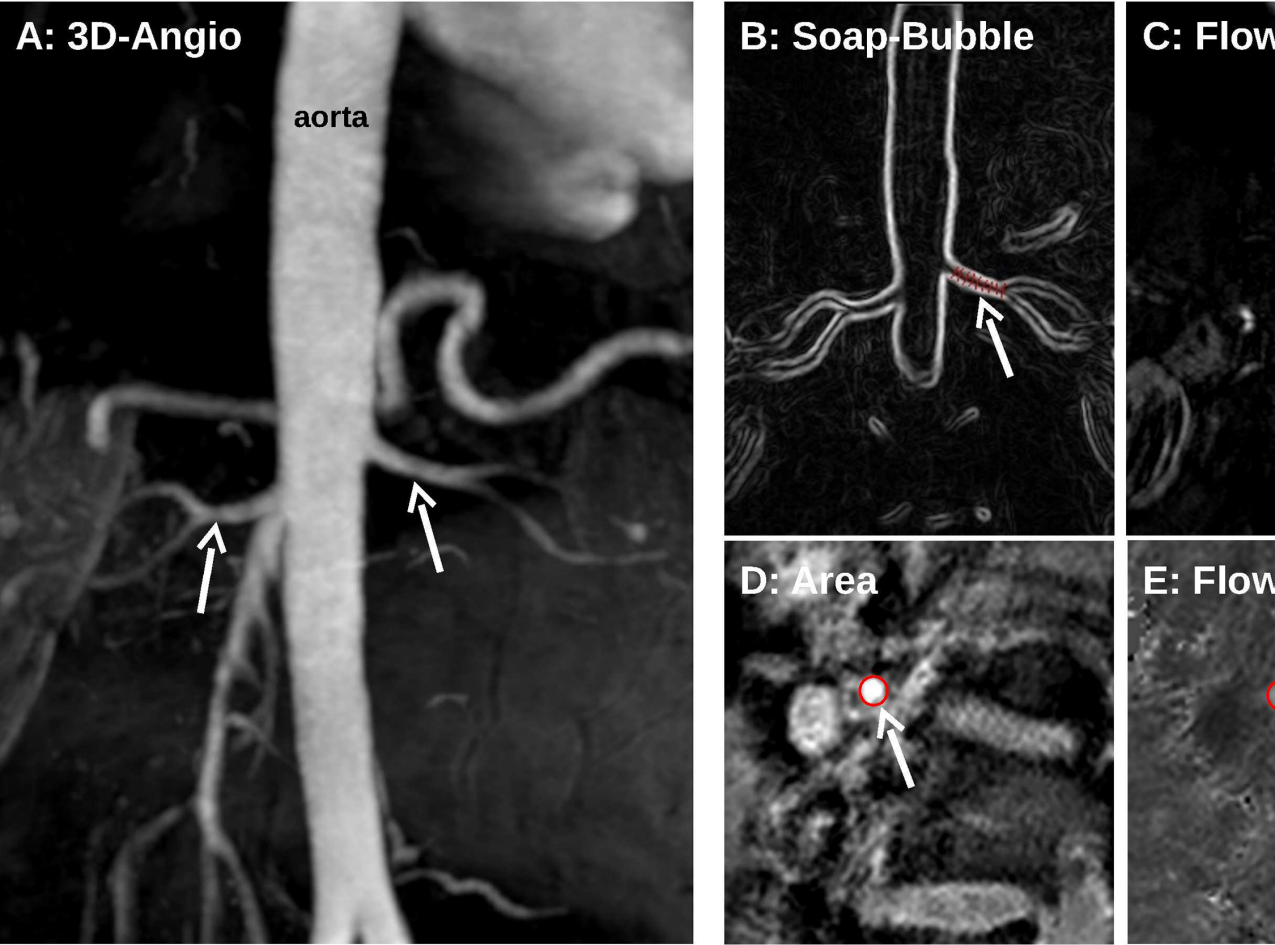
Example of renal artery flow and sharpness evaluation. 3D contrast enhanced angiography (A) images of the abdominal aorta and left (white arrow) and right (dotted arrow) renal artery were postprocessed with the SoapBubble tool, obtaining a 2D representation of the renal artery (B, white arrow indicating the left renal artery)), which was used to quantify renal artery sharpness. Further, in order to evaluate flow parameters, a perpendicular plane to the renal artery was obtained (C demonstrating planning on the left renal artery), allowing the assessment of renal artery area (D) and flow (E), both indicated by red rings (white arrow).

All MR studies were performed in a 3.0 Tesla MR scanner (Ingenia, Philips Healthcare, Best, The Netherlands). The standard anterior and posterior coils were used for signal detection. Images were acquired during breath-holds of 10–15 s using vector electrocardiogram gating. In all patients a standard breath-hold 3D contrast-enhanced MR angiography with a spoiled gradient-echo sequence of both renal arteries was performed after administration of 0.1 mg/kg gadobenate meglumine (Dotarem, Guerbet, Villepinte, France). Typical parameters were TR 4.3 ms, TE 1.4 ms, flip angle 30°, reconstructed voxel size 0.64 x 0.64 x 1.7 mm and number of slices = 92. (Fig 1A and 1D)

In a subset of 17 patients flow measurements of both renal arteries were obtained using through-plane breath-hold phase-contrast MR imaging. The imaging plane was obtained perpendicular to the renal artery, at 10–20 mm from its origin (13.9 ± 2.4 mm for the right renal artery and 12.8 ± 2.7 mm for the left). A gradient-echo sequence with spiral read out (number of interleaves 11, acquisition window 35 ms) with retrospective gating and through plane flow encoding was used. Other parameters were flip angle 20° and voxel size: 0.69 x 0.69 x 8 mm (reconstructed). The encoding velocity value was individually adjusted according to blood flow velocity. (Fig 1B and 1C)

### Post-processing

Renal artery sharpness, a quantitative measure of vessel delineation with higher sharpness values corresponding to better delineation, was quantified from MR angiography using the Soap–Bubble software (SoapBubble Tool V3, Philips Medical Systems, Best, The Netherlands) [14]. Several points were manually selected along the renal artery as visualized in MR angiography, and an automatic 2D representation of the renal artery was obtained (Fig 1E).

From renal artery flow imaging peak velocity, the mean flow, maximal and minimal area, and renal artery distensibility were calculated. Extended MR WorkSpace 2.6.3.5 (Philips Medical Systems, Best, The Netherlands) was used for post-processing. The contour of the renal artery was traced manually on phase-contrast images and was automatically propagated through all phases. A manual correction was performed if necessary. Peak velocity, mean flow, and maximal and minimal area were automatically quantified. Renal artery distensibility was calculated as:

$$Distensibility=\frac{AreaMax-AreaMin}{PP\times AreaMin}$$

where AreaMax and AreaMin refer to maximal and minimal cross-sectional areas, and PP refers to pulse pressure [15].

### Computational fluid dynamics

Three-dimensional MR angiography data were used for the anatomy assessment and phase-contrast MR data were used to set peak systolic flow conditions just at the ostium of both renal arteries. The flow in the reconstructed part of the descending aorta, like flow rates in all other branches (including renal arteries), was calculated assuming Murray law for a relation between flow rate and vessel diameter Q~d^3^ for branching vessels. The anatomy of the aorta was segmented and reconstructed with the software ZIB-Amira (Zuse Institute Berlin, Germany) as described previously [16]. Flow was simulated using ANSYS^®^ Fluent^®^ 14.5 (ANSYS Inc., Canonsburg, PA, USA). A non-Newtonian blood model was applied using an adapted power law model as described earlier [17]. A k-ω SST transition turbulence model assuming turbulence intensity of 5% was used. At the inlet of the aorta the plug velocity profile was taken. At all outlets the outlet boundary condition applying zero diffusion flux for all flow variables and an overall mass balance correction was applied. High quality unstructured volume meshes accounting for ≈1 million cells varying with the volume of the aorta were fabricated with the Gambit^®^ (ANSYS Inc.) following requirements and a mesh independence study. Convergence criteria were set to residual errors <10^−5^.

### Statistical analysis

Statistical analysis was performed using SPSS for Windows (version 19, SPSS Inc., Chicago, Illinois). All continuous parameters are given as mean ± standard deviation (median). Categorical data are summarized as frequencies and percentages. The significance of mean differences between baseline and 6 month follow-up values were tested with the Student T test for paired data (if distribution was normal) or the Wilcoxon test (if normality could not be assumed). The Mann-Whitney test was used to compare changes in outcomes between responders and non-responders. Pearson’s chi-squared test was used to compare categorical data. Linear model analysis was performed to assess the interaction of blood pressure with changes in velocity, maximal area, minimal area, and flow. The Kolmogorov-Smirnov test was used to assess normality. Intra-class correlation coefficient (ICC) was calculated to evaluate intra- and inter-observer variability; an ICC > 0.6 was considered “good” and > 0.7 “excellent” [18]. ICC is given as “ICC (95% confidence interval)”. A p-value < 0.05 was considered statistically significant.

## Results

In total, 32 resistant hypertension patients undergoing RD were included. All patients underwent MR renal artery angiography, corresponding to a total of 64 single renal artery angiograms. No angiographies were excluded. A subset of 17 patients underwent additional flow imaging of both renal arteries. Of the 34 single renal arteries examined, two had to be excluded due to the presence of artifacts. Thus, the final number of flow measurements analyzed was 32. Finally, 20 renal arteries were assessed with computational flow analysis.

Table 1 summarizes the baseline characteristics of our population. Six months after RD, a significant decrease in both systolic and diastolic blood pressure was observed: 155 ± 18 (152) mm Hg at baseline vs. 145 ± 15 (141) mmHg at follow-up, p = 0.001, and 84 ± 10 (84) mmHg at baseline vs. 80 ± 10 (80) mmHg, p = 0.014, respectively. Heart rate was also significantly decreased at follow-up: 70 ± 11 (69) at baseline vs. 66 ± 7 (66), p = 0.022. Renal function was stable 6 months after RD, without significant differences in creatinine value (0.93 ± 0.2 (0.87) mg/dl at baseline vs. 0.96 ± 0.2, (0.91) mg/dl at 6 months p = 0.107) or GFR (81.8 ± 18.8 (81.9) ml/min/1.73 m^2^ before RDN vs. 78.2 ± 17.1 (80.8) ml/min/1.73 m^2^ at 6 months, p = 0.059) in comparison to baseline levels. In the 11 patients in whom the Cystatin-C value was available, no differences were found between the values at baseline and follow-up (0.99 ± 0.16 (0.99) mg/dl vs. 0.99 ± 0.15 (1.1) mg/dl at 6 months, p = 0.755). A significant decrease in the number of antihypertensive agents being taken was observed after RD (4.8 ± 1.5 (4) vs. 4.5 ± 1.5 (4), p = 0.044).

**Table 1:** Baseline characteristics.

Baseline characteristics	Patients (n = 32)
Female gender	9 (28%)
Age (yr)	65 ± 7.5 (67)
BMI (kg/m^2^)	30 ± 4 (30)
Systolic BP (mmHg)	155 ± 18 (152)
Diastolic BP (mmHg)	84 ± 10 (84)
Pulse Pressure (mmHg)	70 ± 11 (69)
Coronary artery disease	16 (50%)
Atrial fibrillation	6 (19%)
Stroke/TIA	3 (9%)
Diabetes mellitus II	13 (41%)
Hyperlipidemia	19 (59%)
Smoking	5 (16%)
Hypertension	32 (100%)
Anti-HT agent medication (n)	4.75 ± 1.5 (4)
ACEI / AT1-blockers	30 (94%)
Renin-inhibitors	3 (9%)
β-blockers	25 (78%)
Calcium Channel blockers	24 (75%)
Diuretics	31 (97%)
Sympatholytics	14 (44%)

BMI: body mass index, BP: blood pressure, Anti-HT: antihypertensive, ACEI: angiotensin converting enzyme inhibitors.

### Renal artery assessment

**Fig 2:**
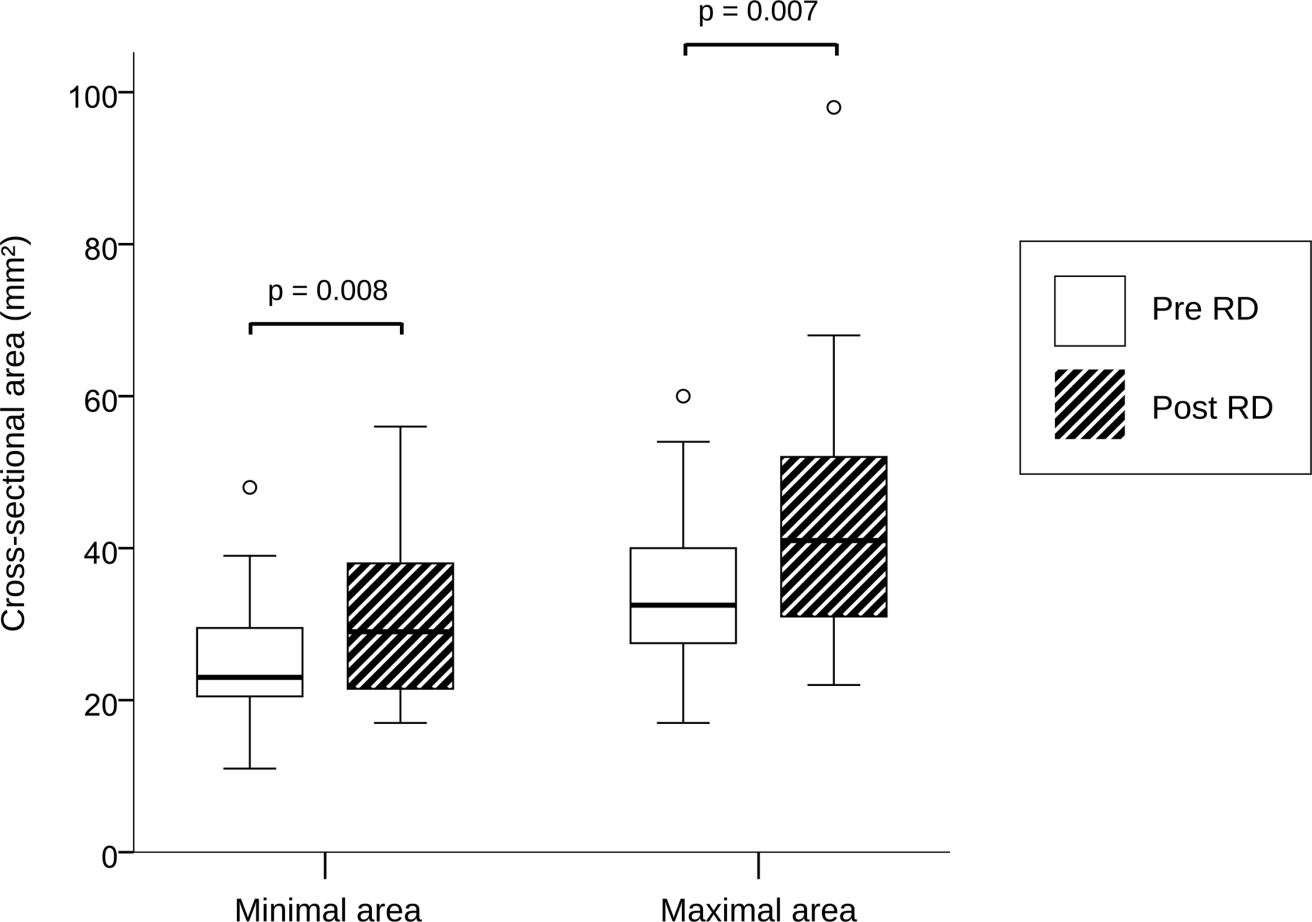
Change in minimal and maximal areas after renal denervation. As shown in this box-plot graph, a significant increase in both minimal and maximal cross-sectional areas 6 months after renal denervation (RDN) was observed.

**Fig 3:**
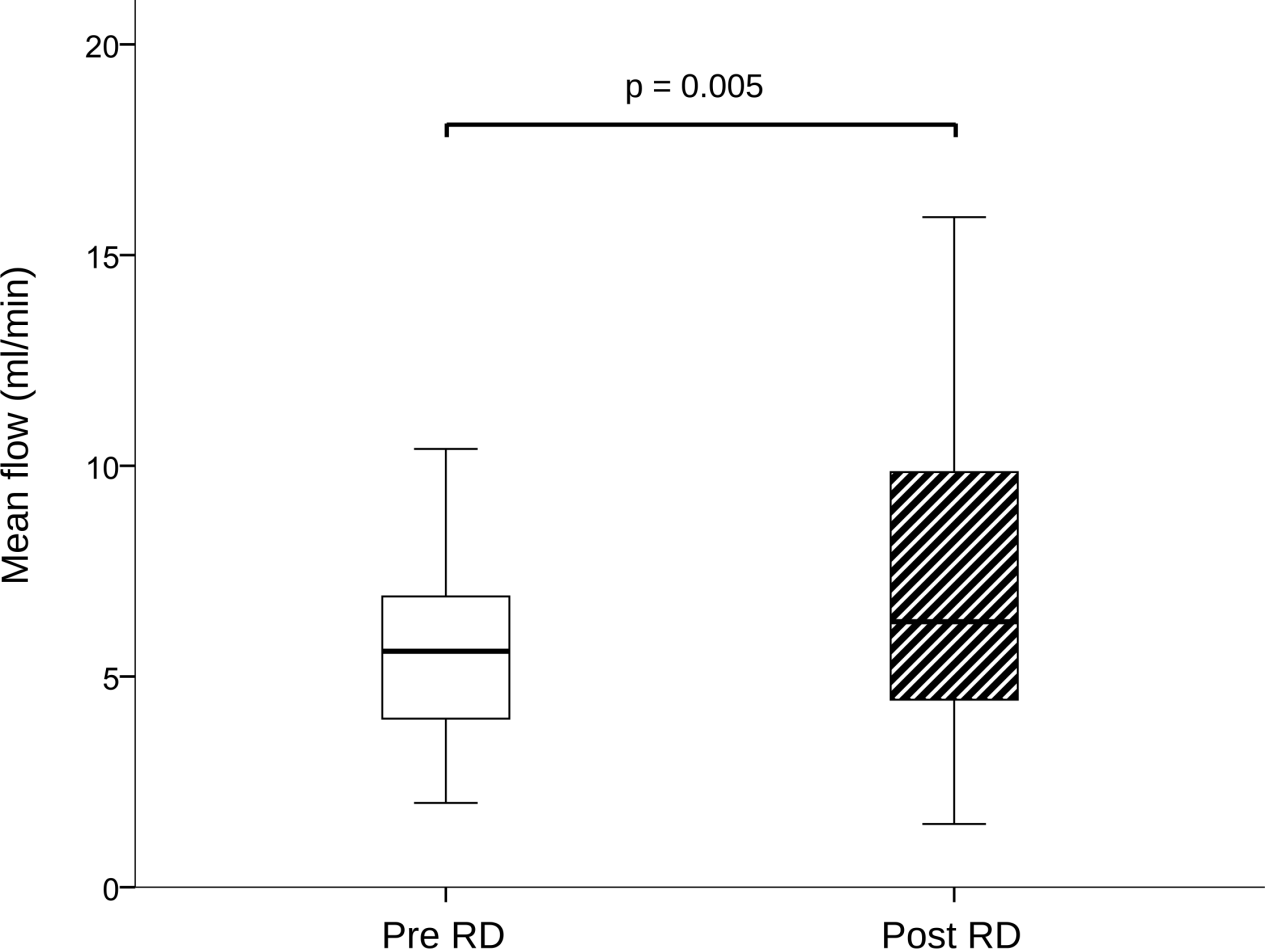
Mean flow change after renal denervation. Renal artery mean flow had increased significantly at follow-up, as shown here.

**Fig 4:**
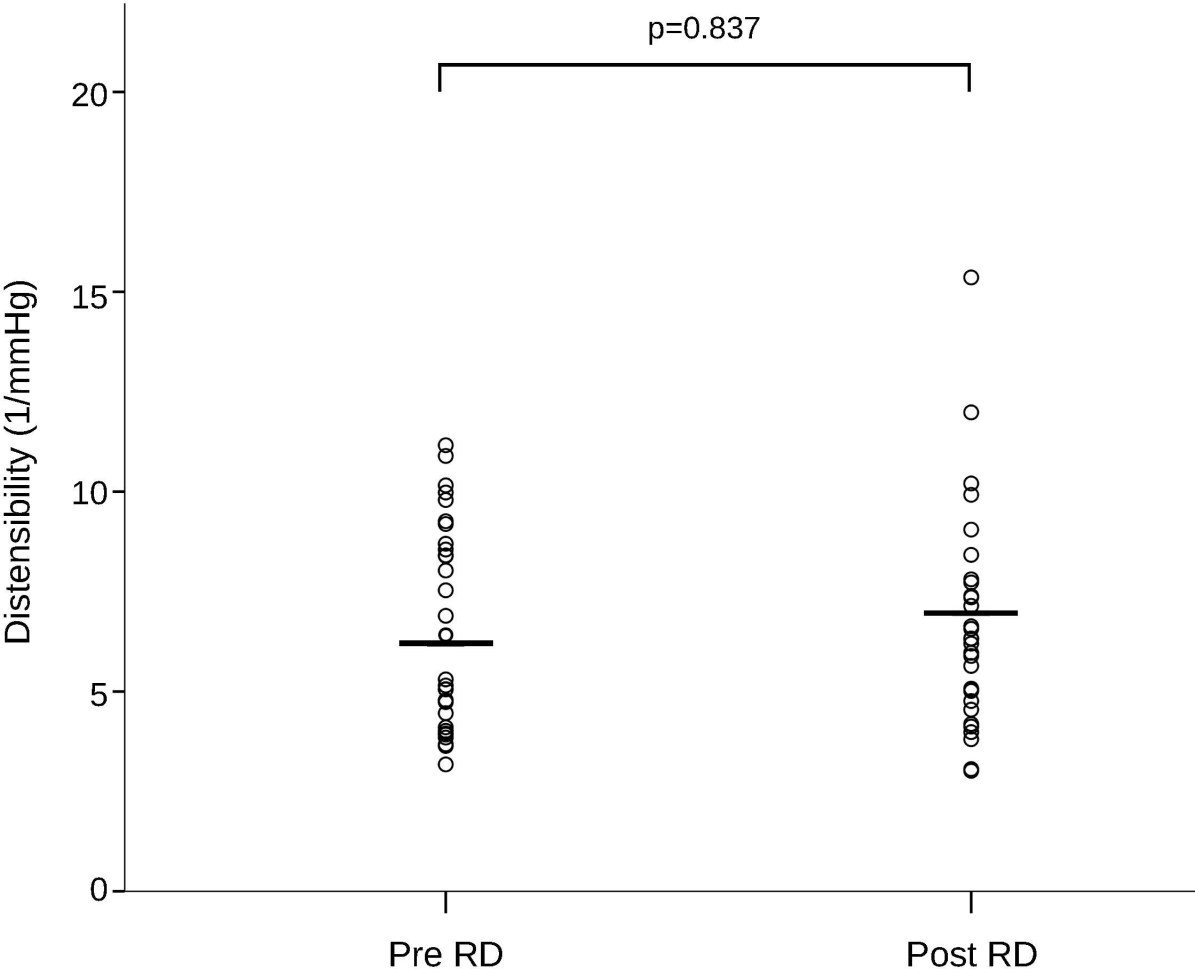
Change in renal artery distensibility after renal denervation. After minimal and maximal cross-sectional areas were obtained, renal artery distensibility was calculated. The distensibility values did not significantly differ after renal denervation as compared to baseline.

**Fig 5:**
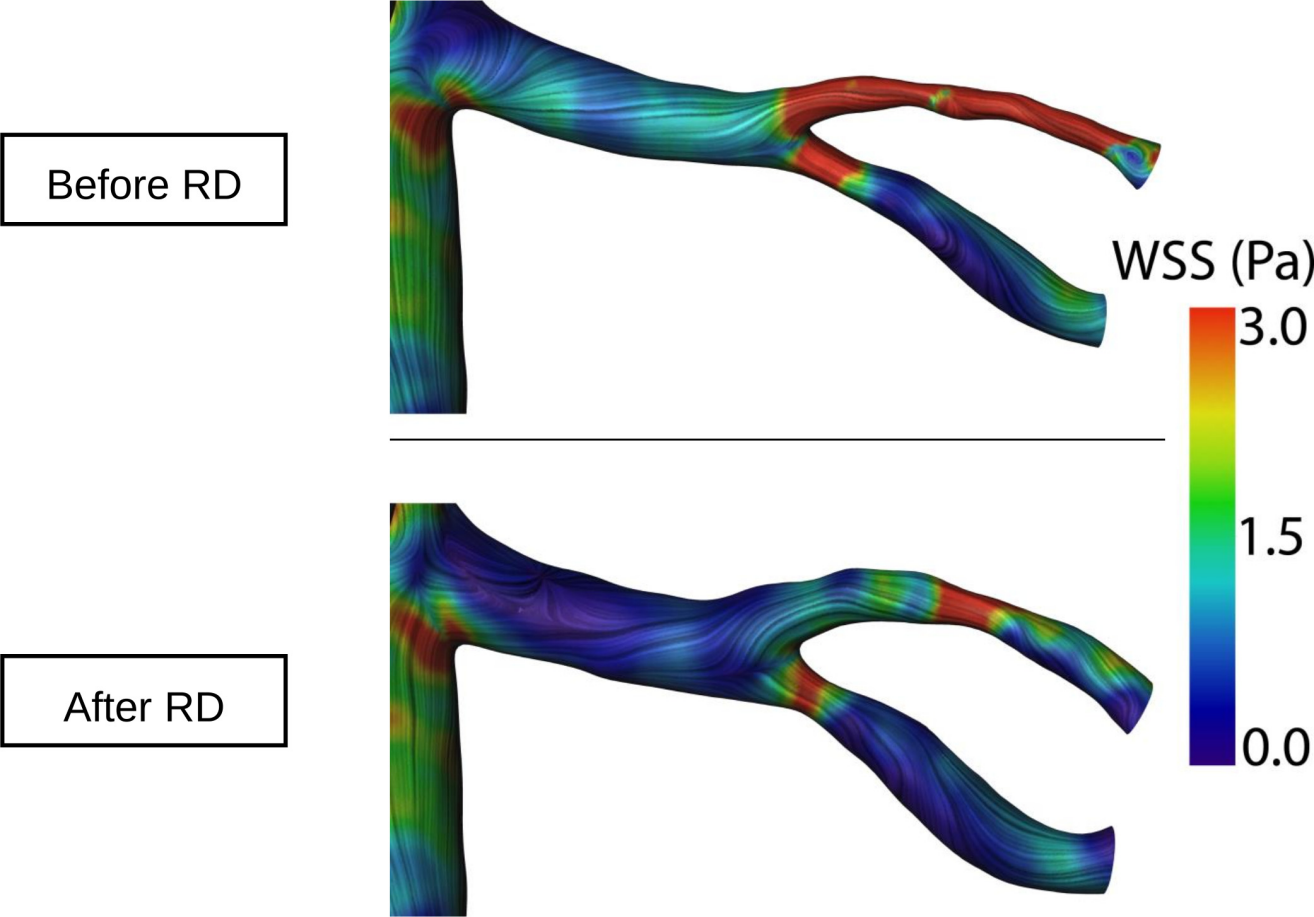
Example of wall shear stress analysis before and 6 months after renal denervation. In this example, the extent of red colored areas, representing higher values of wall shear stress, decreases after renal denervation. Wall shear stress decreased by 25% in the general population.

**Fig 6:**
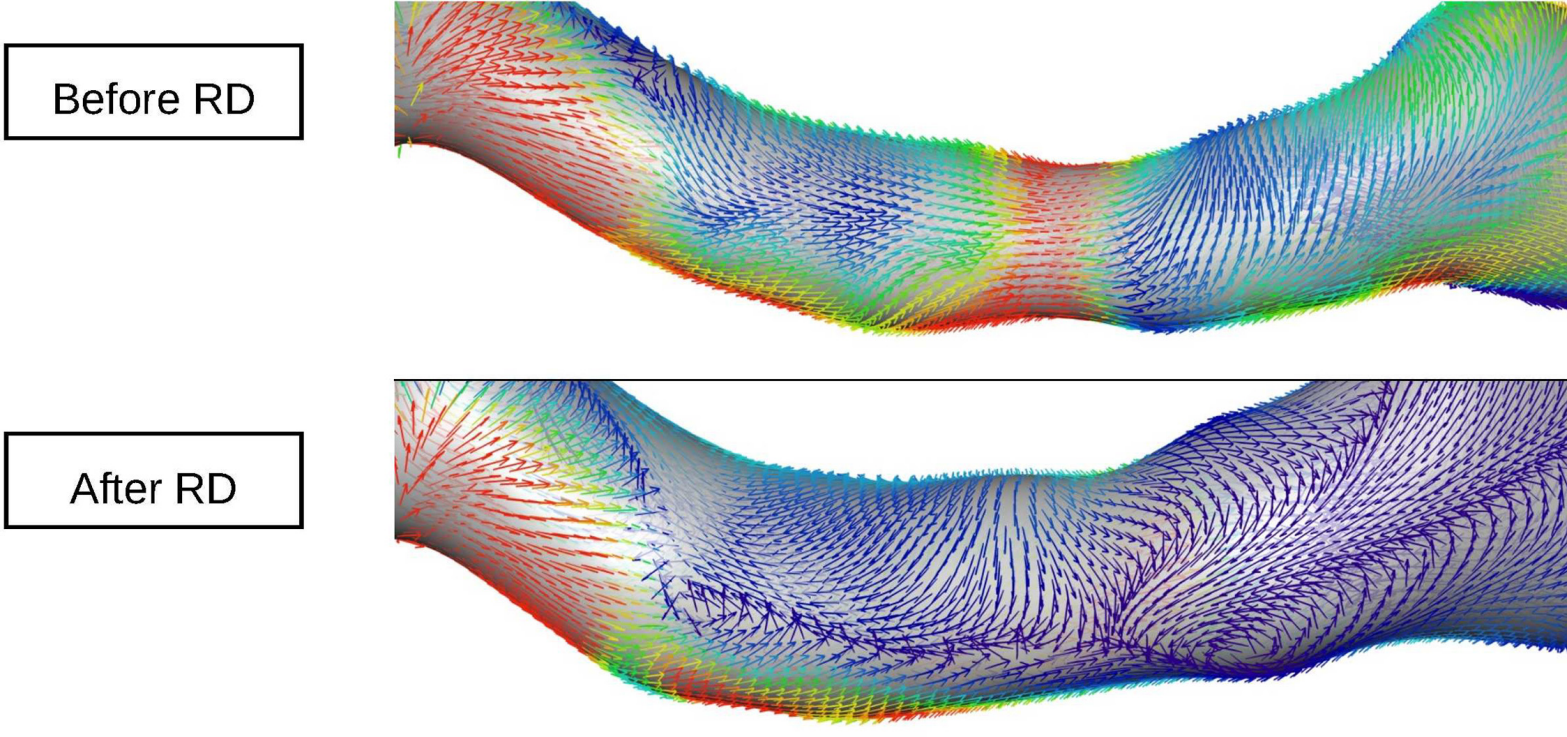
Example of wall shear stress analysis. At 6 month follow-up significantly lower values of wall shear stress (blue) are noted. In addition, enlargement of the renal arteries at follow-up compared to baseline can be observed.

Excellent intra- and inter-observer variability was found in all our measurements (S1 Table). Renal artery sharpness had not significantly changed at follow-up as compared to baseline (48 ± 6.85 (48) % at baseline vs. 47 ± 7.48 (47) % at follow-up, p = 0.399) (Fig 1). Conversely, a significant increase in peak velocity (656.72 ± 178.86 (620) mm/s baseline vs. 767.53 ± 301.55 (704) mm/s at follow-up, p = 0.021), maximal cross-sectional area (34.81 ± 9.51 (32.5) mm^2^ baseline vs. 43.38 ± 16.01 (41) mm^2^ at follow-up, p = 0.007), minimal cross-sectional area (24.81 ± 8.03 (23) mm^2^ baseline vs. 31.09 ± 10.99 (29) mm^2^ at follow-up, p = 0.008), and mean flow (5.8 ± 2.84 (5.6) ml/s baseline vs. 7.1 ± 3.48 (6.3) ml/s at follow-up, p = 0.007) was observed at 6 month follow-up (Figs 2 and 3). No significant changes in renal artery distensibility were found (6.54 ± 2.49 (5.75) 1/mmHg baseline vs. 6.62 ± 2.62 (6.32) 1/mmHg at follow-up, p = 0.837) (Table 2) (Figs 2, 3 and 4). There was no significant interaction between blood pressure and peak velocity, minimal area, maximal area, or renal artery flow in the linear model analysis. The computerized flow analysis demonstrated a significant decrease in wall shear stress at 6 month follow-up in comparison to baseline (1.87 ± 1.23 Pa baseline vs. 1.39 ± 0.78 Pa at follow-up, p = 0.029) (Figs 5 and 6).

**Table 2:** Flow measurements by MR.

	Renal Arteries (n = 32)		
	Pre-RDN	6 months	p
Peak velocity (mm/s)	656.7 ± 178.9 (620.0)	767.5 ± 301.6 (704.0)	0.021
Mean flow (ml/s)	5.8 ± 2.8 (5.6)	7.1 ± 3.5 (6.3)	0.007
Min. area (mm^2^)	24.8 ± 8.0 (23.0)	31.1 ± 11 (29.0)	0.008
Max. area (mm^2^)	34.8 ± 9.5 (32.5)	43.4 ± 16.0 (41.0)	0.007
Distensibility (1/mmHg)	6.5 ± 2.5 (5.8)	6.6 ± 2.6 (6.3)	0.837

Results expressed as mean ± standard deviation (median). Min.: minimal, Max.: maximal. Results correspond to 17 patients and 32 renal arteries in total.

### Responders vs. non-responders

Of the 32 patients included, 15 (47%) were responders to RD; 17 (53%) were non-responders. No difference in number of ablation points was observed between both subgroups: right renal artery 5.5 ± 1.2 (6) points for non-responders vs. 5.8 ± 1.2 (6) points for responders, p = 0.433, and left renal artery 5.9 ± 1.0 (6) points for non-responders vs. 5.9 ± 1.0 (6) points for responders, p = 0.911. Renal artery sharpness did not significantly change after RD, either in responders (48.87 ± 7.62 (48.47) % baseline vs. 46.91 ± 8.21 (47.14) % at follow-up, p = 0.286) or in non-responders (47.77 ± 6.16 (47.01) % baseline vs. 47.27 ± 6.90 (48.42) %, at follow-up, p = 0.632).

Flow measurements were available in 8 responders (14 renal arteries) and 9 non-responders (18 renal arteries). In non-responders, a significant increase in peak velocity, mean flow, and maximal and minimal cross-sectional areas was observed, whereas those same parameters showed a non-significant trend towards an increase in responders. Renal artery distensibility did not significantly change in any of the subgroups (Table 3).

**Table 3:** Responders vs. non-responders.

	Responders (n = 14 renal arteries)			Non-responders (n = 18 renal arteries)		
	Pre- RD	6 months	p	Pre-RD	6 months	p
Peak velocity (mm/s)	732.2 ± 204.4 (694.5)	816.0 ± 387.9 (841.5)	0.272	598.0 ± 134.3 (604.5)	729.8 ± 217.5 (656.0)	0.031
Mean flow (ml/s)	6.6 ± 3.4 (6.0)	7.2 ± 3.8 (5.7)	0.706	5.1 ± 2.2 (5.6)	7.1 ± 3.3 (6.6)	0.002
Min. area (mm^2^)	26.6 ± 9.6 (23.0)	30.9 ± 10.1 (28.5)	0.197	23.4 ± 6.5 (21.0)	31.2 ± 11.9 (30.5)	0.022
Max. area (mm^2^)	37.1 ± 10.8 (35.5)	41.6 ± 11.8 (40.0)	0.146	33.0 ± 8.2 (31.5)	44.7 ± 18.9 (43.5)	0.022
Distensibility (1/mmHg)	6.2 ± 2.1 (5.7)	6.2 ± 2.1 (6.0)	0.975	6.8 ±2.8 (6.1)	6.9 ± 3.0 (6.3)	0.811

Results expressed as mean ± standard deviation (median). Min.: minimal, Max.: maximal. Results correspond to 8 responder patients (14 renal arteries) and 9 non-responder patients (18 renal arteries).

When the percentage of change in renal artery sharpness, peak velocity, maximal area, minimal area, and renal artery flow was compared between responders and non-responders no differences were observed. A trend was shown towards a greater change in the non-responder subgroup (S2 Table).

Finally, when dividing the population according to heart rate response after RD (decrease in heart rate vs. increase or no change in heart rate), peak velocity, mean flow, and maximal and minimal cross-sectional areas showed either a significant increase or a trend towards increase in both subgroups. Renal artery distensibility did not significantly change (S3 Table).

## Discussion

Our results suggest that RD does not have adverse effects at renal artery level. Both renal artery sharpness and distensibility remained stable after 6 months. Furthermore, our study shows that disruption of sympathetic stimuli with RD leads to an increase in renal artery cross sectional areas, which in turn leads to a secondary increment in renal artery peak velocity and flow and a reduction in wall shear stress. The observed effects were found in responders and non-responders to RD.

Several studies have demonstrated the safety of this procedure, which has a low percentage of renovascular complications and is not associated with significant changes in renal function at follow-up [2–4,19]. In addition, RD not only does not impair renal function but has the potential of improving the incidence of albuminuria [5]. Animal studies have failed to demonstrate significant inflammatory signs in the renal arteries at follow-up [20].

However, some authors have reported the development of new onset renal artery stenosis after RD [21–23]. In addition, acute studies with OCT [6,24] demonstrated the presence of post-procedural vasospasm, wall edema and thrombus formation after RD. Overall, the clinical impact of those findings is unknown.

Our study supports the safety data arising from the main RD trials by showing no chronic negative effects of RD on renal arteries. Both renal artery sharpness and distensibility had not significantly changed at 6 month follow-up. Regarding renal artery sharpness, it is a measurement of image quality of the MR angiograms and, as a consequence, of its reliability to detect significant changes in the renal arteries. The fact, that there was no change after 6 months indicates no major damage of the vessel wall, causing decreased delineation of the renal artery wall. Although animal histological data have shown the presence of significant fibrosis in the adventitia and media layers following RD [20,25], our data suggests that such histological changes cause no impairment of renal artery function in humans.

Another finding of our study is the evidence of an increase in renal artery minimal and maximal cross-sectional areas by 25.3% and 24.6%, respectively. This is likely the consequence of disruption of effective sympathetic nerve stimulation with RD and contrasts with the results of a recent CT renal angiography study, in which the authors found no significant changes in renal artery diameter or area at 1 year follow-up [26]. The discrepancy between our results and those of Zhang and colleagues is probably due to the fact that CT angiography, unlike phase-contrast MR, is not a dynamic test and, as a consequence, changes in renal artery area with blood pulse (i.e., maximal and minimal cross sectional areas) cannot be accurately assessed, making the results from two different scans difficult to compare.

We have also demonstrated that the reduction in sympathetic stimuli with RD leads to an increase by 16.9% in peak velocity and by 22.4% in mean flow. This finding is concordant with prior data from animal experiments. In a swine model study it was demonstrated that RD causes an acute increase in renal artery peak velocity and renal blood flow, which persists at follow-up [8]. In the same study, the authors argue that an increase in these parameters could be useful as a marker of effective sympathetic disruption at renal artery level. If such markers were to be used, MR could be employed to perform a non-invasive, radiation-free assessment of renal artery hemodynamics as well as to rule out potential complications of RD at the renal artery level.

Importantly, we found the aforementioned changes in both responders and non-responders to RD. This is concordant with reports of RD showing effects on left ventricular mass and function that are independent of blood pressure reduction [10,27]. Our findings suggest that achieving a reduction in sympathetic stimulation *at the renal artery level* may not be enough to produce a sustained reduction in blood pressure in some patients. Recent human studies have shown that renal nerve density is lower in distal segments of the renal arteries [28]. Lack of effective denervation has been suggested as a cause for non-response [29–31].

Finally, we found a decrease in renal artery wall shear stress, as assessed with computerized flow analysis. Although the decrease in renal artery wall shear stress in our study is consistent with the observed increase in cross-sectional areas, its clinical significance is unclear and was not investigated in the present study. Further works should shed more light on this subject.

## Limitations

Our work is based on a single center study, with a relatively small population. This is particularly true of the flow simulation data which is time-consuming and requires advanced computation. This should be taken into account when interpreting the results, and limits the evaluation of subgroups, although the intraclass correlation coefficient revealed excellent intra- and interobserver reproducibility of our measurements. Our study lacks a control group; therefore, it cannot be ruled out that the changes observed are due to antihypertensive treatment or natural history of the disease. New studies including a higher number of patients and controls are needed to confirm our results. We did not investigate the effects of renal artery anatomy, branching, and number and localisation of the ablation points on our measurements. This should also be further explored. Finally, our follow-up period was limited to 6 months; therefore, the results at later time points could potentially vary and cannot be predicted.

## Conclusions

Our results demonstrate for the first time that RD is not associated with long-term detrimental effects on renal arteries in humans. After RD the sympathetic stimuli in the renal arteries are effectively disrupted, causing vasodilation of the renal arteries, increasing their flow and flow velocity, and reducing wall shear stress. Finally, the clinical relevance of the changes observed in the renal arteries after RD should be investigated in further studies.

## Supporting Information

S1 Table Inter- and intraobserver reproducibility. (PDF)

S2 Table Percentage of change in responders and non-responders. (PDF)

S3 Table Results according to heart rate response. (PDF)
