# Supplementary material for: Effects of Renal Denervation on Renal Artery Function in Humans: Preliminary Study
Source: PLoS One. 2016 Mar 22;11(3):e0150662. doi: 10.1371/journal.pone.0150662 (PMC4803336; doi:10.1371/journal.pone.0150662)
Supplement: S1 Table — (PDF) [file pone.0150662.s001.pdf]

SUPPORTING INFORMATION 1

S1 Table: Inter- and intraobserver reproducibility

	Interobserver	Intraobserver
Peak Velocity	0.993 (0.982-0.997)	0.941 (0.852-0.976)
Mean Flow	0.985 (0.962-0.994)	0.921 (0.801-0.969)
Min. Area	0.948 (0.866-0.980)	0.935 (0.839-0.974)
Max. Area	0.930 (0.822-0.973)	0.953 (0.883-0.981)
Vessel sharpness	0.981 (0.951-0.992)	0.954 (0.885-0.982)

Results expressed as intraclass correlation coefficient (95% CI). Min.: minimal, Max.: maximal.
