# Supplementary material for: Effects of Renal Denervation on Renal Artery Function in Humans: Preliminary Study
Source: PLoS One. 2016 Mar 22;11(3):e0150662. doi: 10.1371/journal.pone.0150662 (PMC4803336; doi:10.1371/journal.pone.0150662)
Supplement: S2 Table — (PDF) [file pone.0150662.s002.pdf]

SUPPORTING INFORMATION 2

S2 Table: Percentage of change in responders and non-responders

	Responders	Non-responders	p
Sharpness change (%)	- 2.3 ± 19.8 (- 1.6)	- 0.7 ± 11.8 (- 1.5)	0.647
Peak velocity change (%)	8.8 ± 33.9 (3.3)	28.3 ± 54.4 (20.5)	0.319
Mean flow change (%)	12.4 ± 50.7 (5.9)	42.3 ± 42.1 (40.3)	0.054
Min. area change (%)	23.9 ± 42.8 (9.2)	38.7 ± 57.7 (27.2)	0.512
Max. area change (%)	18.8 ± 38.5 (7.1)	41.3 ± 69.4 (28.8)	0.419
Distensibility change (%)	7.9 ± 43.0 (- 6.3)	8.2 ± 37.1 (1.4)	0.986

Results expressed as mean ± standard deviation (median). Min.: minimal, Max.: maximal. Results correspond to 15 responder patients (30 renal arteries) and 17 non-responder patients (34 renal arteries) for sharpness, and to 8 responder patients (14 renal arteries) and 9 non-responder patients (18 renal arteries) for the remaining variables.
