# Supplementary material for: Effects of Renal Denervation on Renal Artery Function in Humans: Preliminary Study
Source: PLoS One. 2016 Mar 22;11(3):e0150662. doi: 10.1371/journal.pone.0150662 (PMC4803336; doi:10.1371/journal.pone.0150662)
Supplement: S3 Table — (PDF) [file pone.0150662.s003.pdf]

SUPPORTING INFORMATION 3

S3 Table: Results according to heart rate response

	Heart rate decrease (n = 22 renal arteries)			Heart rate increase or not change (n = 10 renal arteries)		
	Pre- RD	6 months	P	Pre-RD	6 months	p
Peak velocity (mm/s)	674.9 ± 184.1 (628.5)	837.7 ± 308.3 (808.5)	0.006	616.7 ± 168.8 (611.5)	613.1 ± 229.8 (602.0)	0.959
Mean flow (ml/s)	6.3 ± 3.1 (5.6)	7.6 ± 3.5 (6.6)	0.011	4.8 ± 2.0 (5.7)	6.1 ± 3.4 (5.2)	0.202
Min. area (mm²)	26.6 ± 8.4 (23.0)	30.4 ± 10.0 (29.0)	0.135	20.8 ± 5.7 (19.0)	32.6 ± 13.4 (29.5)	0.019
Max. area (mm²)	36.9 ± 10.1 (35.5)	41.1 ± 11.9 (41.0)	0.125	30.2 ± 6.2 (28.5)	48.3 ± 22.7 (43.0)	0.015
Distensibility (1/mmHg)	5.9 ± 2.2 (5.1)	6.1 ± 1.8 (6.1)	0.592	7.8 ± 2.8 (8.8)	7.6 ± 3.8 (7.2)	0.575

Results expressed as mean ± standard deviation (median). Min.: minimal, Max.: maximal.
